# 

**Published:** 1888-10-27

**Authors:** 


### BY INADVERTENCE.

To our great regret by an inadvertence we printed in last week's issue, and sent out in slip as original matter, a little poem entitled "Discharged." We have to thank our contemporary the *St. James's Gazette* for pointing out that "Discharged," is one of the "Hospital Rhymes" from a recently published book by Mr. W. E. Henley. In such circumstances we hope Mr. Henley and our confrères of the Press will accept our sincerest apologies for this unintentional "annexation," which no one can regret more thoroughly than ourselves. "The ethics of quotation may be hazy, no doubt," as our contemporary declares, but we have always endeavoured to secure original matter, and to give full credit to an author whenever his identity can be ascertained.

### The Student's Column.

### ROYAL COLLEGES OF PHYSICIANS AND SURGEONS, EDINBURGH, AND FACULTY OF PHYSICIANS AND SURGEONS, GLASGOW.

The quarterly examinations in Edinburgh for the triple qualification took place in October, with the following results :—

### FIRST EXAMINATION.

Of 65 candidates the following 35 passed : Harvey Francis William de Montmorency, County Carlow : George Edwin Deamer, Lincolnshire ; Herbert Blackburn, Manchester ; Edward Hawkesworth Hackett, Co1k ; Daniel Joseph Murphy, Youghal ; William Herbert Richardson, Manchester ; Henry Ringrose Gore Haynes, Cork ; Edward Whitley Allsom, Liver, ool ; James Philp, Glasgow ; Joseph Frederi k Campbell, Athlone ; George William Johnstone, Edinburgh ; Francis Edward Williams, Cheshire ; John Finlay Mitchell, County Down ; Louis Marie Antoine Enguerrand de La Roche Souvestre, Mau itius ; Tasker Spence Keys, County Donegal ; Charles Alfred Carmalt Jones, London ; Arthur Bailie Francis, Donegal ; Robert Reid, Antrim ; Michael Donovan, County Cork ; Harry Arthur Leonard Howell, Chatham ; Philip Francis Evans, Cork ; Thomas William Waddell, Demerara ; Thom is Lindsay Wilson, Glasgow ; Robert Acton, County Cork ; Caetano Francisco Britto, Goa : John Archibald Campbell, Inverness ; Alexander Geddes Vernor, Musselburgh ; R bert James Black, County Derry ; Thomas Edward Magner, County Cork ; Howard Edward Fellowes, Birmingham ; John Dawson Evelyn, Eynsford ; Arthur Ernest Taylor, Cardiff ; Thomas Canning Hunter, Pontypridd ; Patrick Larkin, Lurgan ; and Walter Fisher, Victoria.

### SECOND EXAMINATION.

Of 77 candidates the following 41 passed : John Clarke Fenwick, Bishopwearmouth ; Robert Emmerson Brown, Durham ; Richard Henry Barber, Worcester ; George Edwin Deam r, Lincolnshire ; Charles Frederick Sixsmith, Ca an ; William Patrick Lonergan, Glasgow ; Godfrey William Waller de Courcy Ba dwin, Tipperary ; John Patrick Walsh, Cork ; Hugh Ferrers Knyvett, Windsor ; James Joseph Foley, Killeagh ; Harvey Francis William de Montmorency, County C rlow ; James Ryan M'Ferran, County Limerick ; Frank Weatherdon Toms, London ; Robert James Collier, Belfast ; George Bridgeford Proctor, Birkenhead ; John Walter Corns, Manchester ; Thomas Finney, Buxton ; James Harvey Martin, Ballynahinch ; Alfred Watson Crooks, Victoria ; Andrew John Douglas Reid, Glasgow ; William Walton Robinson, Chesterfield ; Frederic James Woods, Banbridge ; William Smith, Jamaica ; William Ritchie Main, Lasswade ; Samuel Hickman Davies, Staffordshire ; Richard Snaith J ques, Scarborough ; Joseph O'Brien, County Tyrone ; Robert Tucker Fallon, Westminster ; George Gunn Sinclair, Hamilton ; James Orr, County Antrim ; Joseph Edmund Jones Pegg, Birmingham ; Hugh Shaw, Enniscorthy ; William Fingland, Wavertree, Liverpool ; William James Anderson, Toronto ; Alfred Henry Cheales, Boston, Lincolnshire ; Frank Haden Noott, Dudley ; John Henry Boland, Dundee ; James Francis Curry, Limerick ; Patrick Aloysius Conran, Cork ; George Astin, B rnley ; and Augustus Lower Paliologus, Calcut a.

### FINAL EXAMINATIONS.

Of 84 candidates the following 64 passed, and were admitted L R.C.P.E., L.R.C.S.E., and L.F.P. and S.G. : Mrs. Caroline Keith, Waldhof ; Miss Florence Sorby, Sheffield ; George Edwin Deamer, Lincolnshire ; Frederic Fairburn Armytage, Huddersfield ; M ss Mary Susanna Ackworth, London ; Robert B yle, Glasgow ; Richard Eden Walker, Canada ; William Hamilton Merritt, Canada ; Thomas Warren, Armagh ; Henry Soltau, Exmouth ; Jehangir Jamshedji Cursetji, Bombay ; William Mussen, County Down ; Richard Henry Barber, Worcester ; Edmond William St. Vincent Ryan, Cork ; John Adams, Melbourne ; Eugh William Bailie, County Down ; Robert George Reid, Victoria ; George Ogle Moore, Australia ; Robert Mills Simpson, Ontario ; Charles Edward Salmon, Edinburgh ; Charles M'Leod, Canada ; Frederick Thomas Anderson, India ; Arthur Septimus Thompson, South Shields ; Frederick Naylor Stewart, Newport, Fife ; John Samuel Ledgerwood, County Down ; John Stewart Merrillees, Melbourne ; Richard Patrick Byrne, Cork ; John Stewart Boyd, Renfrewshire ; William Henry Webster, York ; Donald M'Lachlan, Tobermory ; Roger Bernard Burke, Cork ; James Alexander Greig, Edinburgh ; John Ferris, Devonshire ; Willi m Williams, Angelsea ; Francis Malcolm Bovill, London ; Edwin Andrew Cuthbert, Hindmarsh, Calcutta ; George Stevens Pope, Madras ; Alfred Moxon, Rugby ; Miss Elizabeth Simpson Mitchell, Canada ; Miss Nettie Ogilvie, Glasgow ; William Armstrong, Manchester ; Charles James Milligan, Belfast ; Ernest Gerald Robert Whitcombe, India ; Percy Walker Thompson, Toronto ; Robert Rudland, Coventry ; Edmund Lerede Chalke, Madras ; John Henry Brice, Warwickshire ; Harold William Kingcombe Read, Devonshire ; William Arnold Passe, Ceylon ; Joseph Henry Wilson, Co. Cork ; Robert David Prichard Carnarvonshire ; Edward Orr Harrison, Forfarshire ; John Michael O'Dwyer, Cork ; Charles Lawrence Howe, Lancaster ; Luth r Watson, Huddersfield ; Gwilym Evans, South Wales ; John Henry Carson, County Down ; James William Harbinson, Belfast ; John Robert Mason, Lancashire ; William Thomas Blackledge, Lancashire ; Charles William O'Connor, County Limerick ; Francis Murphy, Cork ; John Hurd Gordon, Birmingham ; George Cormick, Tabriz, Persia.

### DENTAL EXAMINATIONS : EDINBURGH.

During the October sittings of the examiners the following gentlemen passed the first professional examination for the licence in Dental Surgery :—

John Thomson Craig, Warwickshire ; Frederick Jones, Lancashire ; Edwin Eli Johnson, Lincolnshire ; Henry Mallett, Devonshire ; John C. Macnamara, Cumberland ; John Stewart, Edinburgh ; and Samuel A. Westerton, London : and the following gentlemen passed the final examination, and were admitted L.D.S., Edinburgh : William Gray, Edinburgh ; Edward John Montague Hodgkinson, London ; and Arthur Turner, Aylesbury.

### Amusements and Relaxation.

### NEW PUZZLE PRIZE SCHEME.

COMMENCED OCTOBER 6th, ENDS DECEMBER 29th, 1888.

On October the 6th a New Prize Scheme was commenced in these columns.

Prizes will be awarded for the best Original Puzzles in Prose or Verse, relating to any of the Social, Philanthropic, or Political topics of the day, and taking the form of Double Acrostics, Anagrams, Square Words, Decapitations, Transpositions, or any other Puzzles which the ingenuity of the competitors may suggest. (*Answers must accompany contributions.*)

*Readers* are requested to send in weekly the Number of Marks they consider each composition to be entitled to, three being the highest score for any one contribution.

Eight Prizes of Standard Books will be given, the choice of the book to be left to the winner.

First, Second, Third, Fourth, and Fifth Prizes for best Original Puzzles, of £1 1s., 15s., 10s. 6d., 7s. 6d., and 5s. each.

First, Second, and Third Prize for Solutions of 15s., 10s. 6d., and 5s. each.

N.B.—All contributions must reach the office, 38, Old Jewry, E.C., not later than the *First Post* on *Thursday* in each week.

### SPECIAL NOTICE.

We have this week received a variety of puzzles in prose and verse all showing more or less ingenuity in their production, but by the non-observance of the rule heading this column, that all *puzzles must relate to some of the social, political, or philanthropic topics of the day*, many competitors are debarred from receiving the number of marks to which they otherwise would be entitled. We feel sure that it is only necessary to call attention once for all to this error to prevent its recurrence. A "Topical Alphabet" embracing all the three given subjects would be an amusing and interesting composition. Who will try his hand at it? It is better to forward one, or even two, really good puzzles each week than several indifferent ones.

### PUZZLES FOR SOLUTION.

**Second Week.**

No. 4. Double Acrostic

Initials downward read will show
A statesman of to-day.
Read finals too and you will know
What office bears his sway.

My first is used to give us light
And chase away the gloom of night.

'Tis in my second that we find
A gem of beauty rare enshrined

My third a man of low degree,
But last of all the tribunes he.

Without my fourth a band is nought,
'Tis heard when there are battles fought.

My fifth reigns in the lonesome night,
But is not visible to sight.

In case for work I should be late
My sixth awakes me up at eight.

An ancient tribe of priestly race,
And Palestine their dwelling-place.

My eighth it is, we fear perchance,
Against our land from traitrous France.

In Russia when the war was thick,
T'was here they nursed the soldiers sick.

A weapon now quite obsolete,
Breech-loaders, rifles, more complete.

The treaty signed, peace once more reigned,
England the rocky fortress gained.

A tale of love and peril too,
But, happily, not always true.

My last the Calendar does hold,
And marks how we are growing old.

Bijou.

No. 5. Original Anagram.

"If poor men die; this sees me hasten." Tinie.

No. 6. Multum in Parvo Puzzle.

I am a word of letters "Ten,"
Begin with "D" and end with "N."
In hospitals I hold no sway,
For I should only cause dismay.
My whole contains ten words as well,
Without transposing you may spell. G. D.

**Marks.**

| Names. | No. of Puzzles sent in Oct 18th. | | No. of Marks Oct. 18th. | | Total of Marks for 1st and 2nd Weeks. |
|---|---|---|---|---|---|
| Patience .......... | 4 | .. | 4 | .. | 9 |
| Lady Clara.......... | 1 | .. | 3 | .. | 6 |
| Tinie .......... | 2 | .. | 4 | .. | 9 |
| Bijou .......... | 1 | .. | 3 | .. | 6 |
| Thanet .......... | 3 | .. | 3 | .. | 10 |
| G. D. .......... | — | .. | — | .. | 3 |
| Daisy .. .......... | 1 | .. | 2 | .. | 4 |
| Salford .......... | — | .. | — | .. | 6 |
| Padre .......... | — | .. | — | .. | 3 |
| Hercules.......... | 5 | .. | 6 | .. | 6 |
| Gretta.......... | 4 | .. | 4 | .. | 4 |
| Dandy Dick ...... | 2 | .. | 5 | .. | 5 |
| Boadicea ........ | 3 | .. | 3 | — | 3 |

(*For Conditions, see last week's* The Hospital.)

